# Death-Associated Protein Kinase Activity Is Regulated by Coupled Calcium/Calmodulin Binding to Two Distinct Sites

**DOI:** 10.1016/j.str.2016.03.020

**Published:** 2016-06-07

**Authors:** Bertrand Simon, Anne-Sophie Huart, Koen Temmerman, Juha Vahokoski, Haydyn D.T. Mertens, Dana Komadina, Jan-Erik Hoffmann, Hayretin Yumerefendi, Dmitri I. Svergun, Petri Kursula, Carsten Schultz, Andrew A. McCarthy, Darren J. Hart, Matthias Wilmanns

**Affiliations:** 1 European Molecular Biology Laboratory, Hamburg Unit, Notkestrasse 85, 22607 Hamburg, Germany; 2 European Molecular Biology Laboratory, Cell Biology and Biophysics Unit, Meyerhofstrasse 1, 69117 Heidelberg, Germany; 3 European Molecular Biology Laboratory, Grenoble Outstation, 71 Avenue des Martyrs, CS 90181, 38042 Grenoble Cedex 9, France; 4 Department of Biomedicine, University of Bergen, Jonas Lies vei 91, 5020 Bergen, Norway; 5 University Grenoble Alpes, Centre National de la Recherche Scientifique-EMBL, Unit of Virus Host-Cell Interactions, 71 Avenue des Martyrs, CS 90181, 38042 Grenoble Cedex 9, France; 6 University of Hamburg Clinical Center Hamburg-Eppendorf, Martinistrasse 52, 20246 Hamburg, Germany

## Abstract

The regulation of many protein kinases by binding to calcium/calmodulin connects two principal mechanisms in signaling processes: protein phosphorylation and responses to dose- and time-dependent calcium signals. We used the calcium/calmodulin-dependent members of the death-associated protein kinase (DAPK) family to investigate the role of a basic DAPK signature loop near the kinase active site. In DAPK2, this loop comprises a novel dimerization-regulated calcium/calmodulin-binding site, in addition to a well-established calcium/calmodulin site in the C-terminal autoregulatory domain. Unexpectedly, impairment of the basic loop interaction site completely abolishes calcium/calmodulin binding and DAPK2 activity is reduced to a residual level, indicative of coupled binding to the two sites. This contrasts with the generally accepted view that kinase calcium/calmodulin interactions are autonomous of the kinase catalytic domain. Our data establish an intricate model of multi-step kinase activation and expand our understanding of how calcium binding connects with other mechanisms involved in kinase activity regulation.

## Highlights

• Members of the DAPK family share a specific basic-loop-mediated dimerization motif
• DAPK2 contains a kinase-mediated and dimerization-regulated CaM-binding site
• Autoregulatory segment CaM binding depends on kinase-mediated CaM binding
• DAPK2 activity is regulated by coupled CaM binding to two different sites

## Graphical Abstract

**Figure:**
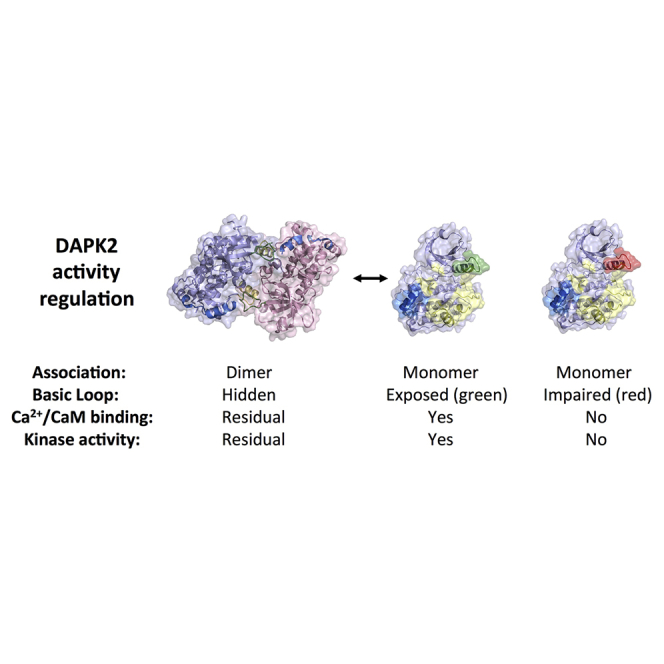

## Teaser

Simon et al. show that human death-associated protein kinase activity is regulated by coupled binding of calcium/calmodulin to two distinct sites, a novel kinase-mediated site and an autoregulatory segment-mediated site, and thus establish a new link to the role of calcium in kinase signaling.

## Introduction

The human kinome comprises more than 500 protein kinases, around 15% of which are predicted to be regulated by calcium/calmodulin (Ca^2+^/CaM) binding (Manning et al., 2002). This type of regulation establishes a crucial link between dose- and time-dependent calcium signaling and central biological processes such as memory potentiation and apoptosis (Harr and Distelhorst, 2010, Wayman et al., 2011). Most predicted Ca^2+^/CaM-dependent kinases comprise an approximately 40-residue autoregulatory domain (ARD) C-terminal to the catalytic kinase domain (CD). For several of these kinases it has been shown that the ARD binds Ca^2+^/CaM with high affinity (Yamniuk and Vogel, 2004). Numerous structural studies of kinase ARD peptide-Ca^2+^/CaM complexes illustrate how both CaM lobes wrap an extended helical ARD segment that is structurally autonomous of the neighboring CD (Dagher et al., 2011, Kuczera and Kursula, 2012). This conclusion was confirmed by a structure of the prototypic calcium/calmodulin-dependent protein kinase II (CaMKII)-Ca^2+^/CaM complex, in which the ARD-Ca^2+^/CaM-binding module is entirely separate from the CaMKII CD (PDB: 2WEL) (Rellos et al., 2010). Surprisingly, in the only other available structure of a kinase-Ca^2+^/CaM complex, death-associated protein kinase 1 (DAPK1), the ARD-Ca^2+^/CaM-binding segment was loosely associated with the DAPK1 CD (PDB: 2X0G) (de Diego et al., 2010). These data suggested that this structure did not represent an activated DAPK1 state and that activity regulation by Ca^2+^/CaM binding could be more complex than previously thought. Thus, the goal of this study was to investigate Ca^2+^/CaM binding to DAPKs to unravel the mechanism of activity regulation by Ca^2+^/CaM binding.

Members of the DAPK family are generally involved in both apoptotic and autophagy pathways (Bialik and Kimchi, 2006) and share significant overall sequence similarity with myosin light chain-related kinases and triple functional domain protein-related kinases (Temmerman et al., 2013). These kinases all have similar patterns of substrate specificity and mechanisms of regulation, with about half of them binding to Ca^2+^/CaM. DAPK1 and DAPK2 bind Ca^2+^/CaM through a closely related ARD, which is inhibited by phosphorylation of an identical ARD residue Ser308 in both kinases (Shani et al., 2001, Shohat et al., 2001). They also contain a basic loop (BL) in the kinase N lobe next to the kinase active site, which has been identified as a DAPK-family-specific signature motif (Tereshko et al., 2001). However, no direct involvement of the loop in DAPK catalysis has been detected (Velentza et al., 2001). Homodimerization of the DAPK1 CD has been shown in solution by mass spectrometry (Zimmermann et al., 2010); however, the functional relevance of this observation and the type of structural arrangement are unknown. For DAPK3, canonical kinase domain-mediated homodimeric assembly via activation loop swapping has been reported (Pike et al., 2008). This type of dimeric assembly is found in a broad range of serine/threonine protein kinases, which require activation by phosphorylation within the CD activation segment (Oliver et al., 2007).

The remaining C-terminal domain organization varies between members of the DAPK family, and distinct modes of kinase activity regulation have been reported for each DAPK (Carlessi et al., 2011, Chen et al., 2010, Jebelli et al., 2012, Llambi et al., 2005). DAPK2 comprises an additional C-terminal tail segment that is predicted to be unstructured, and which has been shown to contribute to kinase activation via another dimerization mechanism that has not yet been fully characterized (Shani et al., 2001). Recent data indicate that this mode of activation can be suppressed by phosphorylation-dependent binding of 14-3-3 scaffold proteins to the C-terminal DAPK2 tail (Gilad et al., 2014) (Yuasa et al., 2015). However, structural studies of the mouse DAPK2 have revealed a CD-mediated homodimeric arrangement that is incompatible with a DAPK2 C-terminal-mediated dimer arrangement (Patel et al., 2011).

Here, we first investigated whether the BL has a role in DAPK activity and/or activity regulation. To address this question, we analyzed and compared new structures of the three most closely related human members of this family: hDAPK1, hDAPK2, and hDAPK3. We found an identical type of dimer arrangement in all three kinases that involves the BL of the CD. By making use of engineered dimer interface mutations in hDAPK2, we found that the BL not only contributes to CD-mediated dimerization but also provides a previously unobserved Ca^2+^/CaM interaction site. When this site is mutated, Ca^2+^/CaM binding and hDAPK2 activity are completely abolished, demonstrating that the established mechanism of activity regulation involving the ARD is not autonomous. The mechanism of dual Ca^2+^/CaM binding unraveled here has features reminiscent of Ca^2+^/CaM trapping in CaMKII, where catalytic activity is regulated via differential Ca^2+^/CaM binding coupled with oligomerization and autophosphorylation. Such conservation of key features in these distantly related kinases indicates that differential Ca^2+^/CaM binding could play an important role in many more Ca^2+^/CaM-regulated kinases and could form a link between kinase activation and Ca^2+^ signaling.

## Results

### Human DAPKs Share a Common CD-Mediated Dimeric Interface

**Figure 1:**
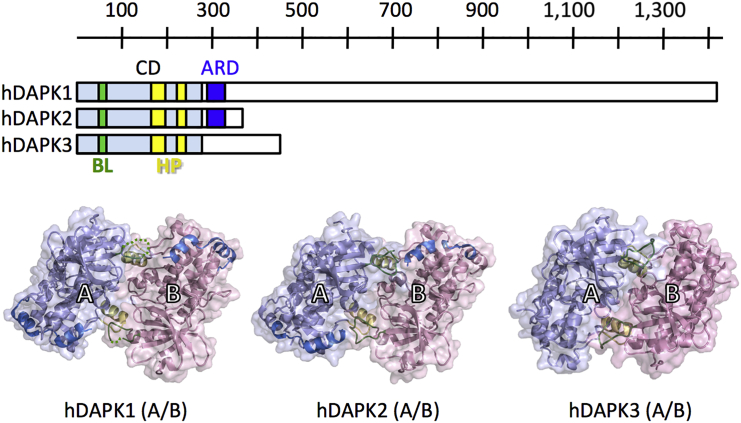
Structures of hDAPK1, hDAPK2, and hDAPK3 Dimers with an Identical Arrangement Upper panel, topology of CD (violet) and ARD (blue) in hDAPK1 (PDB: 2XZS), hDAPK2 (PDB: 2A2A), and hDAPK3 (PDB: 1YRP) sequences. The approximate locations of the BL (green) and the HP (yellow) are also colored. A ruler indicates approximate residue positions. For each structure, the two monomers are depicted as ribbons under transparent surface (violet, blue) showing the basic loop (green), the HP helix αG (yellow), and the ARD (blue). Each monomer is labeled. Dashed lines represent flexible regions that are not supported by electron density (for further details, see Tables S1 and S2). The common dimer arrangement found in all three kinases is equivalent to the starting state on the very left in the cartoon presentation of Figure 7.

To explore the molecular mechanisms involved in human DAPK regulation, we expressed and purified truncated versions of hDAPK1, hDAPK2, and hDAPK3. Both the hDAPK1 and hDAPK2 constructs encompass the CD and ARD segments. Since hDAPK3 has no ARD, the hDAPK3 construct we used was limited to the CD. All three kinases were crystallized and the resulting structures were refined to a resolution of 2.0 Å (hDAPK1), 1.47 Å (hDAPK2), and 3.1 Å (hDAPK3) (Figure 1 and Table 1). The structures presented here all crystallized in the same dimeric arrangement. In this contribution we thought it would be worth investigating this type of dimerization interface further in terms of potentially common functional implications, as such an interface had not yet been previously discovered as a general pattern in different members of the DAPK family.

The hDAPK2 crystal contained two of these dimers per asymmetric unit, referred to as chains A/B and C/D, which provided an additional assessment of experimental error (Figure 1 and Table S1, details described in the legend). The root-mean-square deviation (RMSD) values of superimposed dimers from the different hDAPKs are in the range of 1.2–2.0 Å, demonstrating that the type of dimerization observed in the structures of hDAPK1, hDAPK2, and hDAPK3 is conserved. The dimeric interface areas are in the range of 930–1,600 Å^2^ in size (Figure 1 and Table S2). This area is smaller in the DAPK1 dimer than in the DAPK2 and DAPK3 dimers, as we were unable to model most of the BL region in this kinase due to weak electron density.

**Figure 2:**
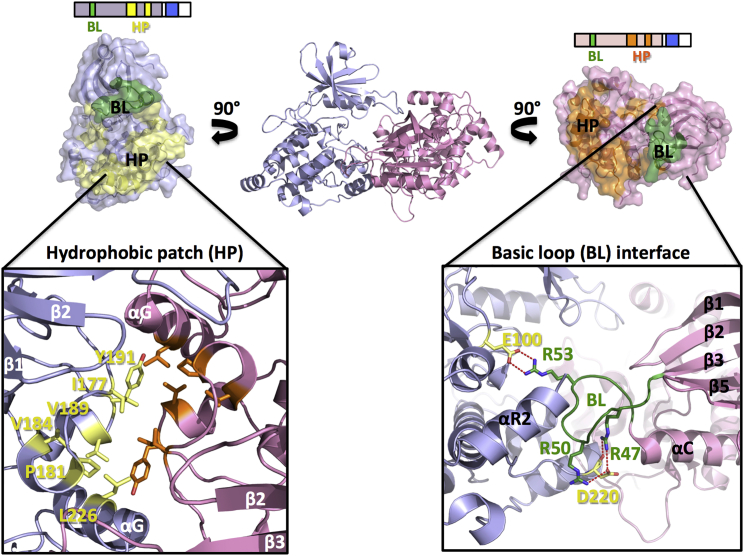
The Homodimeric Assembly of hDAPK2 CD + ARD Involves Two Major Surface Areas Termed “Hydrophobic Patch Interface” and “Basic Loop Interface” The hydrophobic patch (HP) interface and basic loop (BL) interface are colored in orange/yellow and green, respectively. All other color codes are as in Figure 1. The topology scheme from Figure 1 has been included as reference for each monomer. The orientation of the DAPK2 dimer is different from Figure 1 to optimize the view into the dimeric interface. The lower panels zoom into the two interface patches, indicating key interface residues discussed in the text (cf. Figure 3 and Table S2). Interface hydrogen bonds are shown in by red dashed lines.

**Figure 3:**
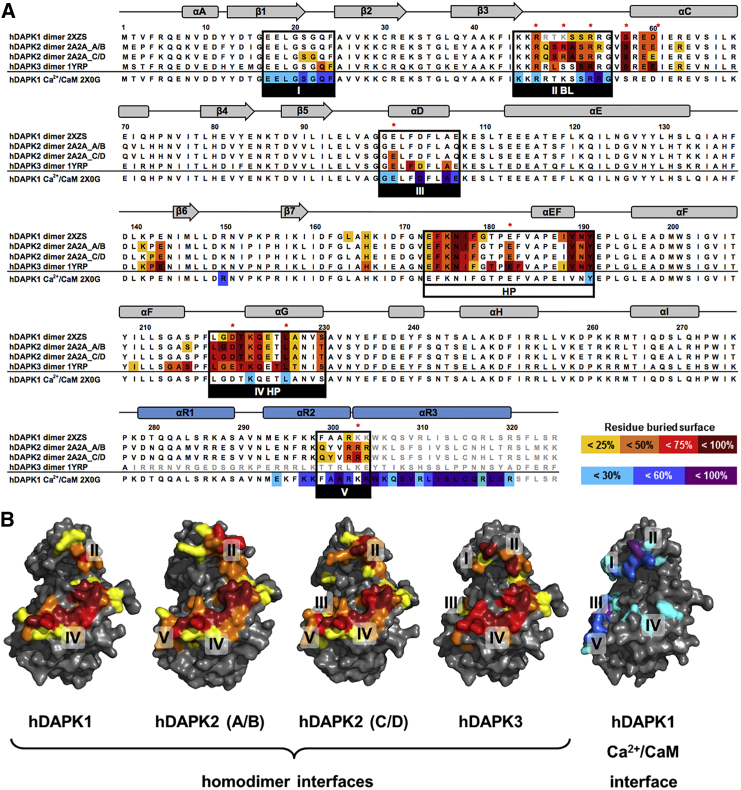
The Structurally Conserved DAPK Homodimeric Interface Overlaps with the DAPK1-Ca^2+^/CaM Interface (A and B) Multiple sequence alignment (A) and dimer surface (B) of hDAPK1 (PDB: 2XZS), hDAPK2 (PDB: 2A2A, chains A/B and C/D), and hDAPK3 (PDB: 1YRP). Secondary structural elements are indicated and labeled according to the conventions established for members of the DAPK family (Temmerman et al., 2013). Interface residues have been identified and characterized with PDBePISA (Krissinel and Henrick, 2007). Residues discussed in the text are labeled with red asterisks. DAPK homodimeric interfaces are colored from yellow to dark red, depending on the level of involvement per residue. The hDAPK1 Ca^2+^/CaM interface is colored from blue to purple. Residues that are not modeled in the respective structures due to lack of interpretable electron density are in gray. Residues that have been mutated and used for functional characterization are labeled in red. Homodimeric interface regions that overlap with the Ca^2+^/CaM-binding interface in hDAPK1 are boxed and numbered I to V. For further details, see Table S2.

In all three DAPK structures, these interfaces comprise two main surface areas. The first one is formed from residues of the substrate-binding region and helix αG from the C lobe of the CD, and the interactions constituting it are mainly hydrophobic, termed the hydrophobic patch (HP) (Figures 2 and 3). This area is highly similar in terms of sequence and rigid structural conformation in all the DAPK structures described here. The second main dimer interface is formed from positively charged residues on the DAPK signature BL segment, which belongs to the most mobile areas in the DAPK structures. In DAPK2, the main residues that contribute to the polar interactions are Arg47, Arg50, and Arg53 as well as the active-site residue Glu100 and Asp220 from the loop preceding helix αG (Figure 2). In the DAPK3 structure, in which the BL residue 50 is a serine instead of an arginine (Figure 3), the most specific interactions are formed by Arg53 and residues C-terminal to the BL from helix αC: Ser57 and Glu60. In DAPK3 there is an additional specific interaction with Glu182, which is the central residue of the PEF/Y motif and a key contributor to DAPK substrate recognition (Figure S1) (Temmerman et al., 2014). By contrast, in the DAPK2 structure, Glu182 is blocked by an interaction with Arg303 from the ARD, which acts as a pseudosubstrate.

In terms of functional implications, CD dimerization in hDAPK1, hDAPK2, and hDAPK3 is likely to inhibit catalytic activity by directly occluding the kinase active site. Furthermore, we observed that this CD dimer interface largely overlaps with that used for interactions with Ca^2+^/CaM, as also seen in the earlier structure of the hDAPK1-Ca^2+^/CaM complex (de Diego et al., 2010). A more detailed analysis revealed five overlapping surface patches that we termed I to V. Two of them, II and IV, coincide with the two main CD dimerization interfaces: the BL-mediated interface (II) and the HP interface (IV). These two patches contribute to more than half of the overall dimer interface area (Figure 3 and Table S2). There are two other segments, I and III, that play prominent roles in Ca^2+^/CaM binding, but they show only minor contributions in DAPK CD dimer interface formation. Finally, there is the N-terminal part of the ARD that participates in both CD dimer formation and the well-established ARD-mediated Ca^2+^/CaM binding, defined as interface segment V. Based on these observations, we investigated whether DAPK dimerization might contribute to the regulation of Ca^2+^/CaM binding. We addressed this question using hDAPK2, as its high-resolution structure provides the most accurate data on dimer interface interactions.

### Structure-Based Design of hDAPK2 Mutants for Functional Experiments

To explore the relevance of the dimeric arrangement, we designed a number of mutants to reduce or abolish dimerization. To ascertain whether hDAPK2 dimerization affects Ca^2+^/CaM binding, we chose residues from segments that contribute to interactions with Ca^2+^/CaM as well as homodimerization (de Diego et al., 2010) (Figure 3). We first targeted the basic loop (Figures 2 and 3) by mutating Arg47, Arg50, Arg53, and Arg54 to glutamate, and we refer to this as the “BL mutant.” We selected this quadruple mutant, as all four BL residues are involved in specific dimer interactions in the hDAPK2 structure. Secondly we selected Asp220, which plays a consistent key role in interacting with residues from the BL (Figure 2) but is not involved in interactions with Ca^2+^/CaM. This residue was changed to a lysine (D220K) (Figure 3). Thirdly we selected Leu226, which also crucially contributes to homodimer interactions with residues from the C-terminal lobe (Figure 2). In contrast to the other residues selected for mutagenesis, Leu226 is oriented toward the predicted kinase substrate-binding site (Figure S2). We mutated it to arginine (L226R), thus matching a defective DAPK2 variant that was previously found in a glioma cancer cell line (Barretina et al., 2012). As a detailed characterization of purified full-length hDAPK2 (hDAPK2 FL) was not possible due to known degradation problems in the C-terminal region (Patel et al., 2011), we used hDAPK2 CD + ARD variants without this tail for further experiments, identical to that used for crystallization.

### DAPK2 Dimer Interface Mutations Lead to Monomerization in Solution

**Figure 4:**
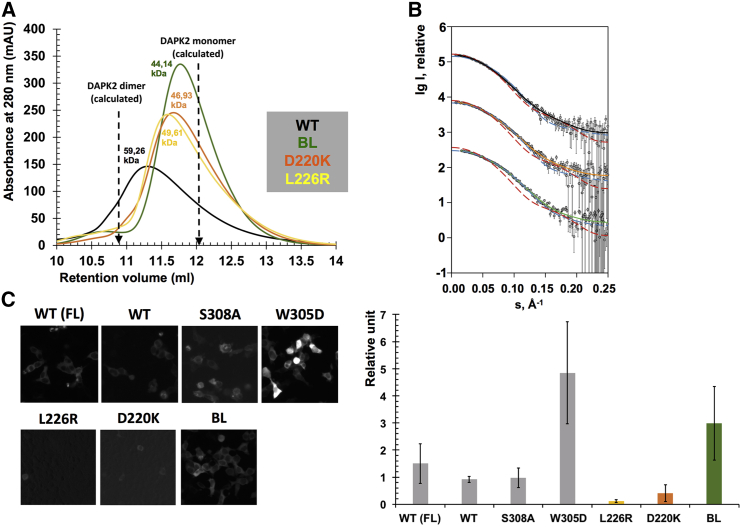
Characterization of hDAPK2 Dimerization Properties (A) SEC analysis of DAPK2 CD + ARD WT (black) and mutants (BL, green; D220K, orange; L226R, yellow). Calculated retention volumes for dimeric and monomeric hDAPK2 are indicated. (B) SAXS experimental curves and model fitting of selected hDAPK2 mutants (for further details, see Table 2 and Table S3). Blue dashed lines, calculated monomer fit; red dashed lines, calculated dimer fit; solid lines (WT, black; D220K, orange; BL, green), calculated equilibrium fit. Y axis values have been manually offset to allow data comparison. Error bars represent the SE of the mean scattering intensities detected at each pixel by the photon counting detector. (C) Dimerization of hDAPK2 mutants by bimolecular fluorescence complementation (BiFC). Left panel: representative images; right panel: quantification of the BiFC readout normalized to protein expression (see Figure S4). Errors bars represent SDs from two independent experiments. Scattering real-space distance distributions are shown in Figure S5.

We first investigated these hDAPK2 mutants by analytical size-exclusion chromatography (SEC) (Figure 4A). Based on the resulting recursive line fitting of calibration standards (Figure S3), we calculated the retention volumes for monomeric and dimeric hDAPK2. Our experimental data on wild-type (WT) hDAPK2 revealed a peak maximum close to the calculated volume of the hDAPK2 dimer. As expected, all hDAPK2 mutants analyzed showed a significant peak shift toward larger retention volumes, indicating loss of dimerization. The strongest effect was seen with the hDAPK2 BL mutant, which yielded close to the expected retention volume of hDAPK2 monomers. The effects we found for the hDAPK2 D220K and L226R mutants are similar to those observed for the hDAPK2 BL mutant. Although the shifts in elution volume were significant, the resolution of the SEC approach was insufficient to resolve separate peaks arising from a mixture of different association states.

To further quantify the oligomeric composition of selected hDAPK2 variants, we performed small-angle X-ray scattering (SAXS) measurements (Figure 4B; for experimental details, see Table 2). For WT hDAPK2, no reasonable fits to either hDAPK2 dimers or monomers were provided by computed scattering curves from different atomic models (for details on software used, see Supplemental Experimental Procedures). The observed discrepancy values in Table S3 indicate that neither of the two association states provides a matching model. However, a significant improvement in fit was achieved when an equilibrium mixture was used to describe the data. The fitting procedure identified a mixture of 51% hDAPK2 monomer and 49% hDAPK2 dimer as the best solution (Table S3; for details on software used see Supplemental Experimental Procedures). By contrast, the hDAPK2 BL mutant was found to be predominantly monomeric (89%). The D220K mutant shows intermediate properties and is well described as an equilibrium mixture of 68% monomers and 32% dimers. In summary, our SEC and SAXS data show that a substantial fraction of hDAPK2 is dimeric in solution under these experimental conditions, and modifications in the conserved interface disrupt dimerization.

### Diverging Dimerization Properties of DAPK2 Mutants Suggest Additional Regulatory Effects

To validate our structural and solution data on hDAPK2 dimerization in a cellular environment, we tested our hDAPK2 constructs using bimolecular fluorescence complementation (BiFC) in HEK293T cells. This assay also allowed us to investigate any effects of endogenous Ca^2+^/CaM binding on hDAPK2 dimerization. To address a possible role of the hDAPK2 C-terminal tail in additional hDAPK2 dimerization (Shani et al., 2001), we first compared the BiFC readout of hDAPK2 FL and C-terminally truncated hDAPK2 CD + ARD (Figure 4C; for analysis of expression levels see Figure S4). The level of dimerization in hDAPK2 CD + ARD was about half that of hDAPK2 FL, which is in agreement with previous data (Shani et al., 2001). To remove any potential effects from the C-terminal tail that is found in hDAPK2 only, we performed all remaining BiFC experiments with truncated hDAPK2 CD + ARD variants.

Next, we tested the possible effects of Ca^2+^/CaM on hDAPK2 dimerization (Figure 4C). As a control, we first used the previously established hDAPK2 ARD mutants S308A and W305D, which have been shown to either promote or substantially reduce Ca^2+^/CaM binding, respectively (de Diego et al., 2010, Shani et al., 2001). Whereas the hDAPK2 S308A variant did not change the BiFC readout significantly, the hDAPK2 W305D mutant increased the BiFC signal more than 5-fold. These data indicate that endogenous Ca^2+^/CaM suppresses hDAPK2 dimerization provided that Ca^2+^/CaM binding is not impaired. The BiFC signal exhibited a markedly reduced level of dimerization for the hDAPK2 D220K and L226R variants, in agreement with the solution data (Figures 4A and 4B). However, the hDAPK2 BL mutant shows an almost 3-fold increase in hDAPK2 dimerization, in contrast to the findings on the same hDAPK2 mutant in solution. In line with our data from the hDAPK2 W305D mutant, showing an increase of DAPK2 dimerization due to impaired Ca^2+^/CaM binding, we hence speculated that there might be a measureable defect in Ca^2+^/CaM binding by the hDAPK2 BL mutant. This is supported by our earlier structural data on the Ca^2+^/CaM complex of the related hDAPK1, in which residues from the BL are indeed involved in specific interactions with Ca^2+^/CaM (Figure 3) (de Diego et al., 2010).

### Defects in the DAPK2 CD Dimeric Interface Affect Its Ability to Bind Ca^2+^/CaM

**Figure 5:**
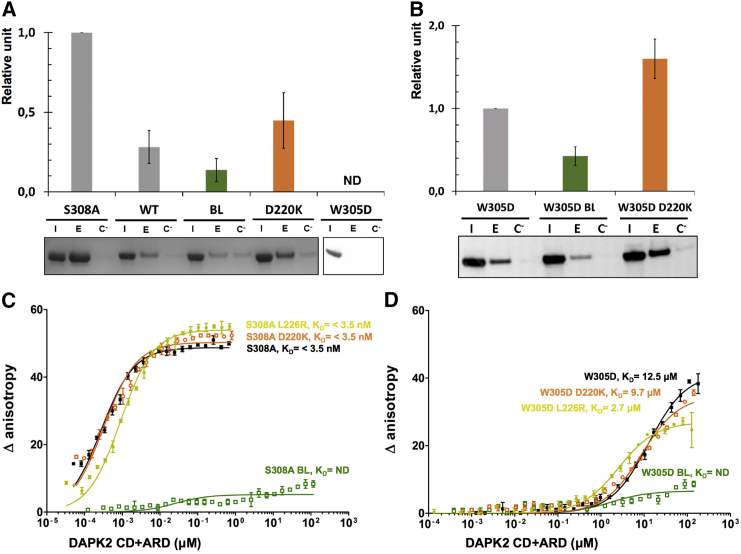
CD-Mediated Ca^2+^/CaM Binding in hDAPK2 (A) CaM pull-down assays of purified hDAPK2 variants monitored by SDS-PAGE Coomassie blue staining. ND, not determined. Data are normalized to the hDAPK2 S308A mutant. Error bars represent one SD from three experimental repetitions. (B) CaM pull-down assays of hDAPK2 variants HEK293T transfected cells monitored by western blot. Data are normalized to the hDAPK2 W305D mutant. I, cell input; E, EGTA-mediated elution on Sepharose 4B-CaM; C, EGTA-mediated elution on Sepharose 4B without CaM (negative control). Relative units were calculated using the average ratio between input and elution band intensities for each mutant over three replicates. Error bars represent one SD from three experimental repetitions. (C and D) In vitro fluorescence anisotropy assays of purified hDAPK2 variants titrated against Ca^2+^/CrAsH-CaM. Error bars represent one SD between the triplicates of a representative experimental curve. K_D_ values were estimated using a non-linear regression assuming one-site specific binding with the software Prism (version 5.0, GraphPad) for three experiments.

To test the possible effects of homodimerization on hDAPK2 Ca^2+^/CaM binding, we designed a series of pull-down assays using two different approaches. First, we assessed the level of hDAPK2 Ca^2+^/CaM binding in solution to purified WT hDAPK2 and the hDAPK2 S308A mutant that can no longer be phosphorylated at position 308. In line with previous data on hDAPK1 (Temmerman et al., 2014), the amount of Ca^2+^/CaM binding by hDAPK2 S308A was around 4-fold higher than that in WT. In the hDAPK2 W305D mutant, Ca^2+^/CaM binding was not detectable under the experimental conditions by direct protein staining (Figure 5A). We then tested the hDAPK2 BL and D220K dimer interface mutants. In the hDAPK2 BL mutant, Ca^2+^/CaM binding was reduced to 46% of the WT protein, whereas we found a 57% increase of Ca^2+^/CaM binding in the hDAPK2 D220K variant.

In subsequent HEK293T cell experiments, we used western blot staining instead of direct protein staining to allow detection of DAPK2 mutants from cell lysate. Under these experimental conditions, the pull-down amounts of WT hDAPK2 and hDAPK2 S308A were too abundant for reliable quantitative analysis due to the much higher sensitivity of the assay. Therefore, we used the hDAPK2 W305D mutant that is compromised in Ca^2+^/CaM binding as a reference and introduced our mutations of interest into this construct (Figure 5B). The hDAPK2 W305D mutant itself produced residual but reliable pull-down data in HEK293T cells. We found a substantial loss of Ca^2+^/CaM-binding ability in the hDAPK2 W305D BL mutant and an almost 2-fold increase of Ca^2+^/CaM binding in the hDAPK2 W305D D220K mutant. Thus, although different references were used for the pull-downs in solution (WT DAPK2) and in HEK293T cells (DAPK2 W305D), both datasets agree in terms of loss of Ca^2+^/CaM binding for the BL mutant and an increase in Ca^2+^/CaM binding for the D220K mutant. Taken together, our findings indicate that although both mutants affect the mode of homodimerization as revealed by our structural and solution data (Figure 4), only the BL mutant is impaired in Ca^2+^/CaM binding. This explains the contrasting behavior of the two mutants in the BiFC assay in the presence of endogenous Ca^2+^/CaM.

### The DAPK2 CD Plays an Essential Role in Ca^2+^/CaM Binding

To further consolidate our findings on matching trends for hDAPK2 Ca^2+^/CaM binding in solution and in cell lines, we measured the Ca^2+^/CaM affinity of different hDAPK2 CD + ARD variants quantitatively in solution by fluorescence anisotropy (FA) with CrAsH-tagged calmodulin (Figures 5C and 5D). We first used the hDAPK2 S308A mutant, in which Ser308 phosphorylation is impaired and thus all sample is expected to bind Ca^2+^/CaM. We estimated its affinity to Ca^2+^/CaM to be <3.5 nM. When this mutant was combined with the hDAPK2 D220K and L226R interface mutants, the binding affinity to Ca^2+^/CaM was still <3.5 nM, demonstrating that neither of these hDAPK2 variants negatively affects Ca^2+^/CaM binding. By contrast, when combined with the BL mutations, the hDAPK2 S308A, BL mutant showed only a residual change in anisotropy that did not allow its binding affinity to Ca^2+^/CaM to be quantified.

We next tested the hDAPK2 W305D mutant that is impaired in ARD-mediated Ca^2+^/CaM binding (Figure 5D), which we used as a reference in the cellular Ca^2+^/CaM pull-down assays described above (Figure 5B). This variant has an estimated Ca^2+^/CaM-binding affinity of 12.5 ± 0.6 μM, which is at least 3,500-fold weaker than that measured for hDAPK2 S308A. This hDAPK2 variant combined with the dimer interface mutants D220K or L226R binds Ca^2+^/CaM with slightly stronger affinity values of K_D_ = 9.7 ± 1.9 μM (D220K) and K_D_ = 2.7 ± 0.1 μM (L226R). The modest improvement in binding affinity is probably due to the increase of monomeric hDAPK2, as observed in our solution and BiFC data (Figure 4). By contrast, no affinity value could be determined for the hDAPK2 W305D, BL variant, which confirms the direct involvement of the BL in CD-mediated Ca^2+^/CaM binding. In agreement with our pull-down data, the FA data also show that impairment of ARD-mediated Ca^2+^/CaM interactions as illustrated by the hDAPK2 W305D mutant substantially reduces the Ca^2+^/CaM-binding affinity by at least three orders of magnitude but does not abolish it. As all known kinase structures in the presence of Ca^2+^/CaM possess an 1:1 stoichiometry of kinase-Ca^2+^/CaM interactions (de Diego et al., 2010, Rellos et al., 2010), these data indicate a coupled CD-mediated and ARD-mediated interaction to the same Ca^2+^/CaM module.

### hDAPK2 Activity Is Regulated by Coupled Ability for Dimerization and Ca^2+^/CaM Binding

**Figure 6:**
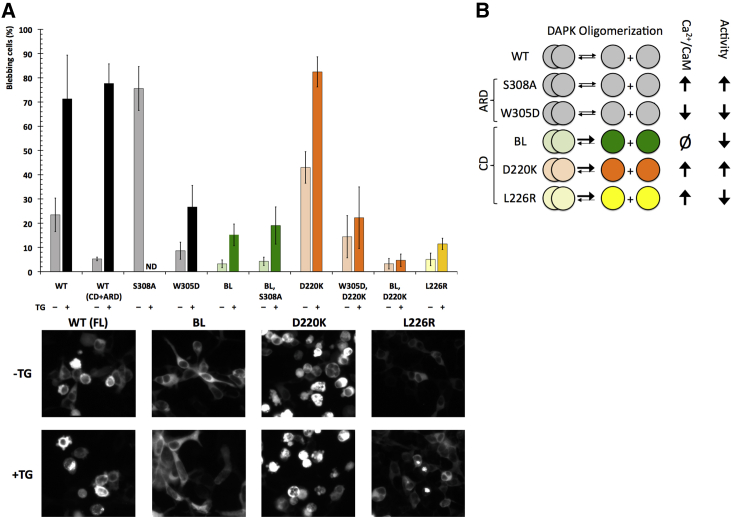
hDAPK2 Blebbing Assay in HEK293T Cells in the Absence and Presence of Thapsigargin (A) Error bars indicate one SD. All experiments were performed in triplicate. Representative images of HEK293T cells expressing hDAPK2 variants are shown below. ND, not determined. (B) Schematic representation outlining our findings on how homodimerization, Ca^2+^/CaM binding, and cellular activity of hDAPK2 are coupled. The thickness of the arrows representing the dimer/monomer equilibrium of different hDAPK2 mutants indicates shifts measured by SEC and SAXS (cf. Figure 4).

Finally, we investigated how the observed coupling of hDAPK2 dimerization and Ca^2+^/CaM-binding ability affects hDAPK2 activity using a well-established blebbing assay in HEK293T cells (Bovellan et al., 2010). In this assay, the hDAPK2-mediated phosphorylation of myosin light chain 2 causes a reorganization of the actin cytoskeleton protruding membrane to form blebs. The number of cells undergoing membrane blebbing can be quantified to reflect hDAPK2 activity (Inbal et al., 2002). All experiments were carried out in the absence and presence of thapsigargin (TG) (Figure 6A), which promotes Ca^2+^/CaM binding (Hoyer-Hansen et al., 2007). As expected, the addition of TG increased blebbing of hDAPK2-expressing cells by more than 3-fold within 5 min. This effect was equally observed regardless of whether hDAPK2 FL or truncated hDAPK2 CD + ARD was used, in line with earlier data (Shani et al., 2001). The activity of the hDAPK2 S308A mutant in the absence of TG is similar to that of WT hDAPK2 in the presence of TG. This indicates that when residue 308 can no longer be phosphorylated and ARD-mediated Ca^2+^/CaM binding can occur, the presence of endogenous Ca^2+^/CaM in HEK293T cells is sufficient to promote unrestrained hDAPK2-mediated blebbing. By contrast, blebbing is largely impaired in the hDAPK2 W305D mutant, even in the presence of TG, as ARD-mediated Ca^2+^/CaM binding is abolished.

The hDAPK2 D220K mutant is as active as WT hDAPK2 in the presence of TG. Moreover, in the absence of TG its activity is even markedly enhanced compared with WT hDAPK2, approaching the effects seen in hDAPK2 S308A. As this hDAPK2 mutant leads to weakening of the hDAPK2 dimer interface without negatively affecting Ca^2+^/CaM binding, we assume that in the D220K variant, CD residues from the BL that are crucial for CD-mediated Ca^2+^/CaM interactions become more accessible to bind endogenous Ca^2+^/CaM in HEK293T cells and promote activity. However, this effect is suppressed when the D220K mutation is combined with the W305D mutation, which is in agreement with our FA data, and suggests that Ca^2+^/CaM binding is dominated by ARD-mediated interactions.

Residual blebbing activity of the hDAPK2 BL mutant remained even below that measured for the hDAPK2 W305D mutant both in the absence and presence of TG, which reconfirms the essential role of the BL in hDAPK2 Ca^2+^/CaM binding. Activity of the hDAPK2 BL mutant could not be restored by combining it with either hDAPK2 S308A or hDAPK2 D220K. This is in agreement with our FA data and demonstrates the essential role of the hDAPK2 BL in Ca^2+^/CaM binding. Similarly, the blebbing activity of the hDAPK2 L226R mutant remained residual. As hDAPK2 Ca^2+^/CaM binding is not affected in this mutant (Figures 5C and 5D), the most plausible explanation is that L226 is involved in protein-substrate binding during catalysis, as this residue is situated close to the substrate-binding site (Figure 3) (Temmerman et al., 2013). In summary, our data demonstrate that DAPK2 catalytic activity is regulated by Ca^2+^/CaM interactions with both the ARD and the BL of the CD, thus coupling the hDAPK2 ability for dimerization and Ca^2+^/CaM binding with hDAPK2 function (Figure 6B).

## Discussion

We have identified and characterized a new type of serine/threonine kinase CD-mediated homodimeric arrangement that is shared by the three most closely related members of the DAPK family: DAPK1, DAPK2, and DAPK3 (Figure 1). Such an assembly has not been found in any other protein kinases and hence seems to be driven by specific DAPK features, such as the BL signature motif. It is different from the previously found dimerization arrangement via swapping of the activation loop between two adjacent kinase domains (Oliver et al., 2007, Pike et al., 2008).

DAPK Ca^2+^/CaM binding and activity is associated with the disassembly of the homodimer into the monomeric state. When investigating the effect of CD monomerization on hDAPK2 activity in more detail, we found a second micromolar-affinity Ca^2+^/CaM-binding site that indeed originates from the BL (Figure 5). Comparison of the specific interactions found in these two sites reveals that the ARD-mediated interactions with Ca^2+^/CaM are much more extensive. This explains why the affinities of the two interaction sites vary by more than three orders of magnitude (Figures 5C and 5D). These data also confirm the unusual versatility of CaM as a scaffold that is able to bind to many proteins with different surfaces and highly adaptive conformations (Yamniuk and Vogel, 2004).

Remarkably, when the BL is mutated such that the CD-mediated Ca^2+^/CaM interaction site is impaired, hDAPK2 Ca^2+^/CaM binding is completely abolished, despite the presence of the nM-affinity ARD-mediated Ca^2+^/CaM interaction site. Our data are thus arguing in favor of a model in which ARD-mediated Ca^2+^/CaM binding is not autonomous of the overall hDAPK2 structure, which was not expected based on previous data mostly on kinase ARD peptide-Ca^2+^/CaM complexes (Dagher et al., 2011, Kuczera and Kursula, 2012, Yamniuk and Vogel, 2004). Indeed, in the hDAPK1-Ca^2+^/CaM complex structure, it was initially shown that ARD-mediated binding requirements for Ca^2+^/CaM are different from isolated ARD peptides due to steric clashes with the hDAPK1 CD (de Diego et al., 2010). Thus, the interpretation of Ca^2+^/CaM-binding data with synthetic ARD peptides requires caution in terms of their relevance under physiological conditions.

As our functional analysis focused on hDAPK2, we next asked to what extent a CD-mediated dimerization module is part of a common regulatory mechanism for members of the DAPK family. In the two DAPK members that are regulated by Ca^2+^/CaM—hDAPK1 and hDAPK2—this mechanism would involve the inhibition of CD-mediated dimerization, the activation of CD-mediated and ARD-mediated Ca^2+^/CaM binding, and a conserved pattern of two phosphorylation sites in the ARD (Ser289, Ser308) with opposing effects on activity regulation (Simon et al., 2015). An analysis of all earlier structures deposited in the PDB reveals that indeed all available DAPK2 structures (PDB: 1WMK, 1Z9X, 1ZWS, 2A27, and 2CKE) show the same type of dimer assembly that we found, confirming the relevance of the data presented here under a broad range of different experimental conditions used for structural analysis.

By contrast, for hDAPK1 there is a substantially higher level of diversity in terms of possible arrangements. Among the 30 deposited DAPK1 structures, we found three with an alternative dimer interface (PDB: 1JKT, 2Y4P, and 3ZXT), which is that of the canonical serine/threonine kinase dimer arrangement by activation segment exchange (Pike et al., 2008). Several other hDAPK1 PDB entries are even monomeric. This is supported by a comparative SEC experiment under the conditions we have established here showing that hDAPK1 tends to be considerably more monomeric than hDAPK2 (Figure S3). An analysis of the level of specific dimeric interface interactions indicates an almost complete lack in the hDAPK1 dimer, compared with those found in hDAPK2 (Table S2). Therefore, we speculate that the inhibition of CD-mediated homodimerization of hDAPK1 may have a less pronounced role in regulating hDAPK1 activity than other family members.

We next investigated whether the complementation of the high-affinity ARD-mediated Ca^2+^/CaM binding by the BL-mediated Ca^2+^/CaM-binding site is generally applicable to other Ca^2+^/CaM-regulated protein kinases. The highly conserved pattern of four positively charged BL residues in DAPK1 and DAPK2 (Figure 3) is suggestive of a common CD-mediated Ca^2+^/CaM-binding site in both these kinases. Our previous structural data on the hDAPK1-Ca^2+^/CaM complex (de Diego et al., 2010) together with the hDAPK2 Ca^2+^/CaM-binding data from this work support this hypothesis.

Moreover, our findings are mirrored in CaMKII in terms of a generally applicable mechanism of dual low- and high-affinity Ca^2+^/CaM binding. In this prototype kinase, the ARD high-affinity Ca^2+^/CaM-binding site is occluded from the dodecameric assembly of the unphosphorylated holoenzyme (Chao et al., 2011). CaMKII phosphorylation of Thr286, located in a loop N-terminal to the canonical ARD high-affinity Ca^2+^/CaM interaction site, leads to a 1,000-fold increase in Ca^2+^/CaM-binding affinity, described as Ca^2+^/CaM trapping (Meyer et al., 1992). This triggers a cascade of phosphorylation of further ARD residues, followed by Ca^2+^/CaM disassembly and acquisition of Ca^2+^-independent kinase activity, which has been described as an autonomous activity state (Stratton et al., 2013). Given that the high-affinity ARD-mediated Ca^2+^/CaM interaction site is conserved in CaMKII and DAPK2 and in both kinases a low-affinity Ca^2+^/CaM-binding state can be detected, it may be interesting to reveal to what extent there are analogies. Conformation-dependent accessibility of known sites for Ca^2+^/CaM binding seems to be a crucial parameter. However, since in the structure of the CaMKII CD + ARD-Ca^2+^/CaM complex the ARD-Ca^2+^/CaM module is entirely separate (Rellos et al., 2010), ultimately this question can probably only be resolved by a still awaited structure of the CaMKII holo-Ca^2+^/CaM complex.

**Figure 7:**
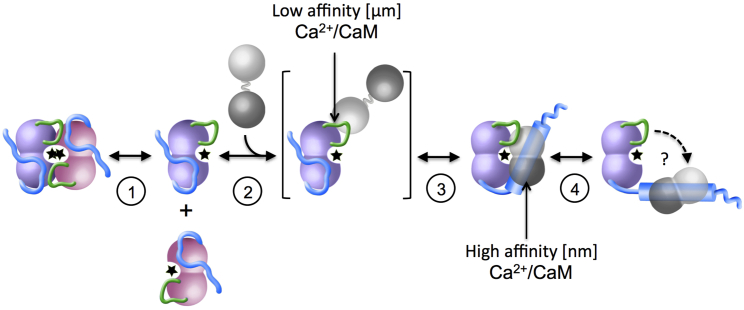
General Mechanism of DAPK Activity Regulation CD-mediated monomerization (step 1), micromolar-affinity CD-mediated Ca^2+^/CaM binding (step 2), and nanomolar-affinity ARD-mediated Ca^2+^/CaM binding (step 3). Step 4—release of the Ca^2+^/CaM-bound ARD module—has not yet been mechanistically investigated and is therefore labeled with a “?”. For reasons of clarity, effects on DAPK activity regulation by ARD phosphorylation are not included in this scheme. Color codes are as in Figures 1 and 2. The DAPK CD active site is indicated with a star. As extracted from available structural data the ARD helix, shown by a cylinder, is formed upon Ca^2+^/CaM binding.

Taking earlier data and those of this contribution together, we propose an extended model of DAPK activity regulation, which so far has been fully demonstrated here for hDAPK2 (Figure 7). It builds on previously established general mechanisms of regulation, including ARD phosphorylation, ARD-mediated Ca^2+^/CaM binding, and conformational switches in the CD region covering helix αD (de Diego et al., 2010, Shani et al., 2001, Simon et al., 2015). In our extended model, the monomeric state of a dynamic dimer/monomer equilibrium is the active form that allows CD-mediated Ca^2+^/CaM binding (Figure 7, steps 1 and 2). This equilibrium may vary between different members of the DAPK family in which dimerization has been observed, as indicated by our structural comparison above. BL-mediated Ca^2+^/CaM interactions are a prerequisite for high-affinity ARD-mediated Ca^2+^/CaM binding (Figure 7, step 3), resulting in a structural arrangement found previously (de Diego et al., 2010). What hinders autonomous ARD-mediated Ca^2+^/CaM interactions in the absence of CD-mediated Ca^2+^/CaM binding at the molecular level is not yet fully understood. This could result from pseudosubstrate-type interactions of the N-terminal part of the ARD with the CD active-site area, as observed in various CD + ARD structures of members of the DAPK family, which may occlude the ARD segment from being accessible for Ca^2+^/CaM binding. Independently from this, Ser308, which serves as one of the key ARD-Ca^2+^/CaM interactions sites, must not be phosphorylated. The last step (Figure 7, step 4), full activation of DAPK through the release of the Ca^2+^/CaM-bound ARD module from the CD surface, is required to allow full access of protein substrates, but this step still needs to be verified by structural data. Based on the structure of the hDAPK1-Ca^2+^/CaM complex (de Diego et al., 2010), Ser289 phosphorylation would inevitably lead to such a fully active state, which is supported by recent data on hDAPK2 (Isshiki et al., 2012). Whether this is accompanied by a conformational change in ARD-bound Ca^2+^/CaM, as observed in several kinase ARD peptide-Ca^2+^/CaM complexes, also remains to be determined.

Our data agree with other established kinase dimerization models that functionally couple dimerization with activity regulation (Endicott et al., 2012). However, whereas dimerization by activation segment swapping in other serine/threonine protein kinases promotes specific activation upon segment phosphorylation (Oliver et al., 2007, Pike et al., 2008), there is no phosphorylation site in the equivalent sequence segment in hDAPK1 and hDAPK2. Our data are restricted to one member of the DAPK family (hDAPK2) and only take the CD and ARD segments into account. All members of the DAPK family investigated here—DAPK1, DAPK2, and DAPK3—have additional domain segments in their C-terminal regions with unrelated oligomerization properties, and an overall model for their involvement in regulation has been proposed (Shiloh et al., 2014).

The logical next step will be to investigate our findings in the context of the respective full-length DAPK proteins. From a potential drug discovery point of view, these additional sites for activity regulation could provide promising starting points for the rational design of novel inhibitors.

### Significance

About 15% of all human protein kinases are estimated to be regulated by calcium-bound calmodulin (CaM), demonstrating the significance of connecting calcium-dependent and phosphorylation-dependent signaling processes. Common to most of those kinases is that CaM is bound to a small, separate, and highly conserved autoregulatory segment C-terminal to the kinase domain. Previous data indicated that CaM binding to this segment is modular and independent from the neighboring kinase domain, raising questions regarding how this could be coupled with other known mechanisms of kinase activity regulation. In this study, we searched for mechanistic principles of connecting different types of kinase activity regulation such as phosphorylation and oligomerization by investigating CaM-regulated members of the DAPK family. In DAPK2, which we took as a model, we discovered a novel CaM-binding site mediated by a common DAPK family signature loop with low-micromolar affinity. As this loop is also involved in DAPK2 kinase dimerization, monomerization promotes CaM binding. Surprisingly, when this kinase-mediated CaM-binding site is impaired there is also no CaM binding to the well-established autoregulatory segment, challenging previous assumptions on modular, autonomous CaM binding to regulate kinase activity. Our data generate an integrated four-step model in DAPK activity regulation: (1) kinase domain-mediated homo-dimerization, (2) kinase-mediated CaM binding, (3) autoregulatory segment-mediated CaM binding, and (4) DAPK phosphorylation of specific residues sites. Comparison of our findings with other prototype CaM-dependent protein kinases is indicative that dual and coupled CaM-mediated kinase activity regulation could present a general principle of kinase activity regulation. The discovery of the kinase-mediated CaM-binding site opens novel opportunities for targeting this site by small-molecular inhibitors and subsequent drug discovery approaches.

## Experimental Procedures

The hDAPK1 construct comprising the CD and most of the autoregulatory domain (CD + ARD, residues 1–312) was expressed from a pET9a-derived vector encoding an N-terminal hexahistidine tag with a tobacco etch virus (TEV) cleavage site in *Escherichia coli* BL21(DE3) strain induced with 0.1 mM isopropyl β-D-1-thiogalactopyranoside (IPTG) and grown at 25°C overnight. The cells were lysed using a microfluidizer in 50 mM Tris-HCl (pH 8.0), 300 mM NaCl, 5 mM imidazole, 5 mM β-mercaptoethanol, protease inhibitor cocktail (Complete, Roche Life Science) and 1/1,000-fold diluted benzonase (Novagen). The lysates were centrifuged at 76,500 × *g* for 30 min and the supernatant incubated with 500 μl of Ni-nitrilotriacetic acid (NTA) resin (Qiagen) overnight at 4°C. The resin was collected by centrifugation at 200 × *g* for 1 min and resuspended in 5 ml of lysis buffer. It was then loaded on gravity columns and washed first with 10 volumes of buffer A (50 mM HEPES-NaOH [pH 8.0], 1,000 mM NaCl, 10 mM imidazole, and 1 mM DTT) followed by 20 volumes of buffer B (50 mM HEPES-NaOH [pH 8.0], 100 mM NaCl, 50 mM imidazole, and 1 mM DTT). The protein was eluted with buffer B containing imidazole in steps of 100, 200, 300, and 400 mM. The purified fractions were centrifuged for 10 min at 20,817 × *g* at 4°C and 5 mg of protein was incubated with 50 μg of hexahistidine-tagged TEV protease for 8 hr at room temperature. The protein was dialyzed to remove imidazole in buffer (50 mM Tris-HCl [pH 8.0], 100 mM NaCl, and 1 mM DTT) for 1 hr at room temperature and the TEV protease was removed with a second Ni-NTA column. Finally, the protein was subjected to SEC with S200 10/300 Gl (Amersham, GE Healthcare).

For hDAPK2 and hDAPK3, the respective cDNA was amplified from their pcDNA vector by PCR (Shani et al., 2001). The products, corresponding to hDAPK2 comprising the CD and autoregulatory domain (CD + ARD, residues 10–330) and hDAPK3 comprising the CD (CD, residues 1–274), were subcloned into the Gateway system (Invitrogen) vector pDEST-15, with an N-terminal glutathione S-transferase tag. The recombinant proteins were expressed in the *E. coli* Rosetta (DE3) pLysS strain at 20°C, inducing with 0.4 mM IPTG overnight. The cell pellet was resuspended in 20 mM HEPES (pH 7.5) and 150 mM NaCl, after lysis and centrifugal clarification. The recombinant protein in the supernatant was bound to glutathione Sepharose-4B (GE Healthcare). After washing with 10 column volumes of lysis buffer, elution was performed by TEV protease cleavage. Both proteins were further purified by SEC using an S75 10/600 Gl column (GE Healthcare).

For analytical SEC, SAXS, and CaM pull-down and FA assays, hDAPK2 CD + ARD was amplified from its pcDNA vector (Shani et al., 2001) by PCR and cloned into pETM14 for expression in *E. coli* BL21 (DE3) star pRARE2. Cells were grown in Terrific Broth medium containing 30 μg/ml kanamycin and 34 μg/ml chloramphenicol at 37°C to a maximal optical density of 1.0–1.2 at 600 nm. Protein expression was induced by 0.1 mM IPTG at 20°C for 20 hr. Cells were harvested and sonicated in 50 mM HEPES-NaOH (pH 7.5), 250 mM NaCl, 5% (v/v) glycerol, 5 mM CaCl_2_, 10 mM imidazole (pH 7.5), 0.2 mM tris(2-carboxyethyl)phosphine (TCEP), 1 mg/ml DNAseI, 10 mg/ml lysozyme, and protease inhibitors (Pefabloc SC, Roche Life Science). The lysates were centrifuged at 24,000 × *g* for 30 min at 4°C and the supernatants were filtered and applied to an Ni-NTA resin (Qiagen). Proteins were eluted with 50 mM HEPES-NaOH (pH 7.5), 250 mM NaCl, 5% (v/v) glycerol, 5 mM CaCl_2_, 0.1 mM TCEP, and 200 mM imidazole. The hexahistidine tag was cleaved by 3C-protease at 4°C for 20 hr in 50 mM HEPES-NaOH (pH 7.5), 250 mM NaCl, 5% (v/v) glycerol, 5 mM CaCl_2_, 0.1 mM TCEP, and 10 mM imidazole. The cleaved tag was removed using Ni-NTA resin (Qiagen). The proteins were further purified by SEC, using an S75 10/600 Gl column (GE Healthcare) in 50 mM HEPES-NaOH (pH 7.5), 250 mM NaCl, 5% (v/v) glycerol, 5 mM CaCl_2_, and 0.1 mM TCEP. Selected fractions were collected and concentrated to a maximum of 7 mg/ml before flash-freezing in liquid nitrogen.

Analytical SEC was used to assess the level of dimerization of hDAPK2 CD + ARD variants. For each experiment, 100 μl of protein sample at concentrations between 2.5 and 7 mg/ml were loaded on an S75 10/300 Gl column (GE Healthcare) at a flow rate of 0.2 ml/min at 4°C in 50 mM HEPES-NaOH (pH 7.5), 250 mM NaCl, and 0.1 mM TCEP. The column was calibrated using Bio-Rad gel-filtration standards (#151-1901) according to the manufacturer's recommendations. The DAPK1 CD + ARD construct used for SEC was expressed and purified as previously described (Temmerman et al., 2014).

The hDAPK1 CD + ARD construct (1–312) was crystallized at a concentration of 6 mg/ml in 0.2 M magnesium acetate tetrahydrate, 0.1 M sodium cacodylate (pH 6.5) with 10% (w/v) polyethylene glycol (PEG) 8000, using the hanging-drop vapor diffusion method. The hDAPK2 CD + ARD construct was crystallized at a concentration of 6 mg/ml using the sitting-drop vapor diffusion method. The solution used for crystallization contained 0.2 M lithium sulfate, 0.1 M Tris-HCl (pH 8.5), 25% (w/v) PEG 4000, and 15% (v/v) glycerol. The hDAPK3 CD construct was crystallized at a concentration of 5 mg/ml by the hanging-drop vapor diffusion method. The solution used for crystallization consisted of 0.1 M Tris-HCl (pH 9.0), 0.2 M acetate, 30% (w/v) PEG 6000, and 10 mM DTT. The coordinates and structure factors were deposited in the PDB under the accession codes PDB: 2XZS (DAPK1), PDB: 2A2A (DAPK2) and PDB: 1YRP. Details of X-ray structure determination are described in Supplemental Experimental Procedures.

Further biophysical and functional experiments were carried out with hDAPK2. Details of analytical SEC experiments, SAXS, BiFC, calmodulin pull-down assays, in vitro FA assays, and cellular blebbing assays are described in Supplemental Experimental Procedures.

## Author Contributions

B.S., P.K., D.J.H., A.S.H., and M.W. designed the experiments. B.S., A.S.H., K.T., J.V., H.D.T.M., D.K., J.E.H., H.Y., and A.A.C. performed and analyzed the experiments. B.S. and M.W. wrote the manuscript. D.I.S., C.S., D.J.H., and M.W. supported the work.

## Figures and Tables

**Table 1:** X-Ray Data Collection and Refinement of hDAPK1, hDAPK2, and hDAPK3 Structures

Protein Kinase	hDAPK1	hDAPK2	hDAPK3
PDB ID	2XZS	2A2A	1YRP

**Data Collection**			

X-ray beamline	ESRF, ID14-4	EMBL/DESY, BW7A	EMBL/DESY, X11
Wavelength (Å)	0.9765	0.9206	0.81
No. of reflections	46,826	200,389	10,171
Completeness (%)	95.6 (89.9)	94.8 (93.4)	97.2 (95.5)
Redundancy	3.6 (3.5)	2.5 (1.9)	3.1 (3.0)
Resolution range (Å)	50–2.0 (2.1–2.0)	40–1.47 (1.52–1.47)	20–3.1 (3.2–3.1)
*R*_sym_ (%)	7.5 (33.8)	4.9 (42.4)	9.8 (33.6)
<*I*/σ>	11.5 (3.5)	11.1 (1.9)	11.3 (3.7)
Space group	P2_1_	P1	P2_1_
Unit cell edges (Å)	56.3, 49.6, 130.5	55.3, 60.7, 98.7	53.6, 60.9, 88.0
Unit cell angles (°)	90.0, 94.5, 90.0	92.2, 103.5, 94.3	90.0, 92.2, 90.0

**Refinement**			

Resolution range (Å)	43–2.0 (2.05–2.00)	20–1.47 (1.51–1.47)	20–3.1 (3.2–3.1)
*R*_cryst_/*R*_free_ (%)	18.5/22.5	15.0/20.7	24.5/26.8
No. of protein atoms	4,675	9,884	4,490
No. of heterogen atoms	2	61	8
No. of solvent atoms	243	1,371	0
Mean *B* value (Å^2^)	30.8	23.6	47.2
RMSD bond lengths (Å)	0.024	0.017	0.006
RMSD bond angles (°)	1.825	1.599	1.24
Ramachandran values favored/allowed (%)	96.9/3.1	95.4/3.6	90.3/7.7

Data in parentheses correspond to the highest-resolution shell.

**Table 2:** SAXS Data Collection and Derived Parameters for DAPK2 and Mutants

	WT	D220K	BL
**Data Collection Parameters**			

Instrument	EMBL P12 (PETRA-III)		
Beam geometry (mm^2^)	0.2 × 0.12		
Wavelength (Å)	1.24		
*s* range (Å^−1^)^a^	0.003–0.45		
Exposure time (s)	0.045·20		
Sample concentration (mg/ml)	2.0–15.0	2.0–15.0	2.0–15.0
Temperature (K)	283		

**Structural Parameters**			

*I*(0) (cm^−1^) (from *p*(*r*))	0.027 ± 0.001	0.025 ± 0.001	0.021 ± 0.001
*R*_g_ (Å) (from *p*(*r*))	26.4 ± 0.2	25.9 ± 0.2	23.4 ± 0.2
*D*_max_ (Å)	81 ± 5	90 ± 5	75 ± 5
Dry volume (10^3^ Å^3^) calculated^b^	45		
Contrast (Δρ × 10^10^ cm^−2^)	2.704		
Molecular mass *M*_r_ (kDa) (from *I*(0))	41 ± 5	37 ± 4	32 ± 3
Molecular mass *M*_r_ (kDa) (from Porod volume [*V*_p_/1.7])	59 ± 6	52 ± 5	46 ± 5
Calculated monomeric mass *M*_r_ (kDa)^b^	37.3		

**Software Employed**			

Primary data reduction	Automated pipeline (Blanchet et al., 2015)		
Data processing	ALMERGE, AUTORG, DATGNOM, DATPOROD		
Computation of model intensities	CRYSOL, FFMAKER, OLIGOMER		

a Momentum transfer |*s*| = 4πsin(θ)/λ.

b Calculated from the sequence using www.basic.northwestern.edu/biotools/proteincalc.html.

## Accession Numbers

Structural coordinates were deposited in the PDB (www.wwpdb.org) under the accession codes PDB: 2XZS (DAPK1), PDB: 2A2A (DAPK2), and PDB: 1YRP (DAPK3). The SAXS data and models were deposited at the Small-Angle Scattering Biological DataBank (SASBDB, www.sasbdb.org) under the accession codes SASDB52 (hDAPK2 CD + ARD WT), SASDB62 (hDAPK2 CD + ARD D220K), and SASDB72 (hDAPK2 CD + ARD BL).

## Supplemental Information

Document S1. Supplemental Experimental Procedures, Figures S1–S5, and Tables S1–S3

Document S2. Article plus Supplemental Information
