# Supplementary material for: Death-Associated Protein Kinase Activity Is Regulated by Coupled Calcium/Calmodulin Binding to Two Distinct Sites
Source: Structure. 2016 Jun 7;24(6):851–61. doi: 10.1016/j.str.2016.03.020 (PMC4906247; doi:10.1016/j.str.2016.03.020)
Supplement: Document S1. Supplemental Experimental Procedures, Figures S1–S5, and Tables S1–S3 [file mmc1.pdf]

Structure, Volume *24*

Supplemental Information

Death-Associated Protein Kinase Activity Is Regulated by Coupled Calcium/Calmodulin Binding to Two Distinct Sites

Bertrand Simon, Anne-Sophie Huart, Koen Temmerman, Juha Vahokoski, Haydyn D.T. Mertens, Dana Komadina, Jan-Erik Hoffmann, Hayretin Yumerefendi, Dmitri I. Svergun, Petri Kursula, Carsten Schultz, Andrew A. McCarthy, Darren J. Hart, and Matthias Wilmanns

Supplemental material

Supplemental experimental procedures

Crystallization, X-ray data collection and structure determination

Crystals of hDAPK1 were transferred into a solution with identical crystallization conditions, containing 20% [v/v] glycerol in addition, and were subsequently flash frozen in liquid nitrogen. X-ray data to 2.0 Å resolution were collected on beamline ID14-4 at the European Synchrotron Radiation Facility (ESRF), Grenoble, France. X-ray data were integrated and scaled using MOSFLM (Leslie, 2006) and SCALA (Evans, 2006). Phases were determined by the molecular replacement method using available DAPK1 CD coordinates (Tereshko et al., 2001) as a search model with PHASER (McCoy et al., 2007). Several rounds of manual building were performed with COOT (Emsley et al., 2010) and structure refinement with REFMAC (Murshudov et al., 1997). MOLPROBITY (Chen et al., 2010) was used for model validation.

A single hDAPK2 (CD+ARD) crystal was directly picked and cryo-cooled under a stream of liquid nitrogen (100 K). X-ray diffraction data were collected to 1.47 Å resolution on the EMBL/DESY DORIS beamline BW7A, Hamburg, Germany. X-ray diffraction data of the hDAPK3 CD construct were collected at 100 K to 3.1 Å resolution on the EMBL/DESY DORIS beamline X11, Hamburg, Germany. The hDAPK2 and hDAPK3 X-ray data were processed and scaled with XDS (Kabsch, 2010) and XDSi (Kursula, 2004). Structures were solved with the program MOLREP (Vagin and Teplyakov, 2010), using the coordinates of hDAPK1 CD (Tereshko et al., 2001) as a model. The structure was refined with REFMAC (Murshudov et al., 1997) and ARP/wARP (Perrakis et al., 1999). The software O (Jones et al., 1991) was used for model building and analysis.

Crystallographic X-ray data and structure refinement statistics are summarized in Table 1. All figures were prepared with Pymol (Schrodinger, Version 1.7.2.2)

Small-angle X-ray scattering

X-ray solution scattering data were collected on the EMBL/DESY PETRA beamline P12, Hamburg, Germany, using a PILATUS 2M pixel detector (DECTRIS, Switzerland) with 20 frames of 50 ms exposure time. Solutions of all hDAPK2 constructs were measured while flowing through a temperature-controlled capillary at 10°C in 50 mM HEPES-NaOH buffer (pH 7.5), 250 mM NaCl, 5mM CaCl2, 0.25 mM TCEP, 5% [v/v] glycerol. Each construct was measured at a concentration of 2 mg/ml. Based on a comparison of successive frames, no detectable radiation damage was observed. Intensities were placed on absolute scale relative to water and molecular masses (MMs) of solutes were estimated from the extrapolated forward scattering I(0).

The program OLIGOMER was used to model potential multicomponent mixtures of species in solution (Konarev et al., 2003), where volume fractions corresponding to each component (monomeric and dimeric hDAPK2) were determined by OLIGOMER using a non-negative least squares procedure, where the scattering intensity of a mixture of particles is expressed in terms of additive contributions from the intensities $I_k(s)$ of each component:

$$I(s) = \sum_{k=1}^{K} v_k I_k(s)$$

where K is the number of components and v_k are the corresponding volume fractions. OLIGOMER determines the volume fractions yielding the best fits to the experimental data. Form factors of input PDB files were calculated from PDB code 2A2A, using the program FFMAKER (Petoukhov et al., 2012). Direct fits of monomeric hDAPK2 and dimeric hDAPK2 from PDB code 2A2A were computed with CRYSOL (Svergun et al., 1995). The SAXS data and the oligomeric model equilibrium were deposited at the Small-Angle Scattering Biological Data Bank (SASBDB, www.sasbdb.org) under the accession codes: SASDB52 (hDAPK2 CD+ARD WT), SASDB62 (hDAPK2 CD+ARD D220K) and SASDB72 (hDAPK2 CD+ARD BL).

Bi-molecular fluorescence complementation assay

For bi-molecular fluorescence complementation (BiFC) assays, both hDAPK2 FL (residues 10-370, UNIPROT Q9UIK4) and hDAPK2 CD+ARD (residues 10-330) were cloned into pBiFC-VN (C-terminal Venus I152L residues 1-154) with a 12-residue linker (GTGGGGSGGGGS), and pBiFC-VC (C-terminal Venus residues 155-end) with a 19-residue linker (GTRPACKIPNDLKQKVMNH) vector (Addgene). HEK293T cells were grown

in Dulbecco's modified essential medium (GIBCO) supplemented with 10% fetal calf serum and 1% penicillin streptomycin. HEK293T cells were seeded on Nunc™ Lab-Tek™ II Chamber Slide™ System 8-well plates 4 hours before transfection. Cells were transfected with 0.3 µg DNA for each BiFC construct and 1.5 µl of Lipofectamine 2000™ (Invitrogen) according to manufacturer instructions.

Live cells were imaged 16 hours post transfection with a 25-fold magnification on an AxioObserver Z1 (Carl Zeiss MicroImaging) inverted fluorescence microscope. Images were processed with ImageJ (1.47v, Wayne Rasband, NIH, USA). Cell boundaries were evaluated using fluorescence threshold above background. The total fluorescence over the cell surface was measured on ten images per well. Average intensities of fluorescence by surface unit were used to compare the different hDAPK2 variants. Two experiments with separate samples, for which each was measured twice, were performed for each variant and all values were normalized to WT hDAPK2 FL. Expression levels were assessed by western blotting using anti c-Myc primary antibody for pBiFC-VN constructs (EMBL Protein Expression and Purification Core Facility, Heidelberg, Germany), anti HA primary antibody for pBiFC-VC constructs (Sigma #H3663), anti-GADPH primary antibody (Loading control, Abcam #9484) followed by a horseradish peroxidase (HRP)-conjugated anti-mouse secondary antibody (Sigma #A9917) and was detected using a HRP detection kit (Thermo scientific, Super signal West pico #34087). Expression levels were evaluated by measuring band intensities with Image J (1.47v, Wayne Rasband, NIH, USA) and were corrected for loading using GADPH band intensities. Expression levels were defined as average band intensities of both VN (c-Myc) and VC (HA) construct relative to the wild-type full-length protein present in each experiment. Average intensities of fluorescence by surface unit of each hDAPK2 variant were corrected relative to the expression level determined by western blotting.

Calmodulin pull-down assay

Calmodulin pull-downs were carried out both with E. coli recombinant protein variants and HEK293T cells expressed protein variants. For expression in HEK293T cells, three 25 cm2 flasks of cells were transfected with 8 µg of pBiFC-VN-hDAPK2 FL variants, using 24 µl of polyethylenimine (PEI) at 1 mg/ml (Sigma Aldrich). Cells were grown in Dulbecco's modified essential medium (GIBCO) supplemented with 10% fetal calf serum, reduced to 2% during the 6 hours of transfection, and 1% penicillin streptomycin. After 24 hours of transfection, cells were lysed in 0.2% Triton lysis buffer consisting of 20 mM HEPES-NaOH (pH 7.2), 0.2% (v/v) Triton X-100, 250 mM NaCl, 2 mM CaCl2, 1mM benzamidine, 1 mM phenylmethanesulfonyl fluoride (PMSF), 1 mM TCEP, 0.1 mM ethylenediaminetetraacetic acid (EDTA), 1X protease inhibitor mix (Pefabloc SC®, Roche life science). In the assays with recombinant hDAPK2 variants 26 µg pure protein was added to 30µl calmodulin Sepharose 4B (GE Healthcare) pre-washed in lysis buffer and incubated for 2 hours at 4°C with gentle rotation. In the assays with HEK293T cell lysate, 0.25 mg lysate was added instead. The resin was pelleted by centrifuging at 500 g for 3 minutes at 4°C, and then washed several times with lysis buffer. Calmodulin-bound proteins were eluted by the addition of 30 µl lysis buffer supplemented with 4 mM ethylene glycol tetraacetic acid (EGTA) for 10 minutes at 4°C with gentle shaking. Then, 20 µl of the eluate were mixed in the sample buffer (Nupage®, LDS sample buffer, Invitrogen) at 2.5% β-mercaptoethanol and boiled at 75°C for 5 minutes. Input (2 µg E. coli recombinant pure protein, 15 µg HEK293T cell lysate), eluates (20 µl), and eluates from binding controls using glutathione Sepharose 4B (GE Healthcare) were resolved by SDS-PAGE. E. coli recombinant pure protein was detected using Coomassie-based staining. HEK293T cells expressed protein was detected by western blotting using anti c-Myc primary antibody (EMBL Protein Expression and Purification Core Facility, Heidelberg, Germany) and a HRP detection kit (Thermo scientific). Band intensities were evaluated by measuring band intensities in ImageJ (1.47v, Wayne Rasband).

In vitro fluorescence anisotropy assay

For fluorescence anisotropy measurements, calmodulin was labeled with the small organic dye CrAsH, which is a bisarsenite derivative of carboxyfluorescein (Griffin et al., 1998) It spontaneously reacts with cysteines, preferentially in proteins with a tetracysteine motif (CCPGCC) forming a β-loop (Adams et al., 2002) . The fluorophore was prepared according to established protocols (Cao et al., 2006; Rutkowska et al., 2011). Calmodulin containing an N-terminal FLAG tag and a tetracysteine tag for CrAsH labeling (MDYKDHDGDYKDHDIDYKDDDDKRSFLNCCPGCCMEPSQAS), as well as a C-terminal hexa-histidine tag, which was used for affinity purification, was cloned in a pBAD vector and expressed in E.coli BL21 (DE3) AI cells. The bacteria were grown in 50 ml Terrific Broth medium at 37°C until an optical density of 0.6 at 600 nm, after which expression was induced with 0.02% L-arabinose overnight. Cells were harvested by centrifugation at 4000 rpm over 20 minutes, resuspended in PBS (137 mM NaCl, 2.7 mM KCl, 12 mM NaH2PO4) lysis buffer, 1 mM PMSF and 5 mM imidazole-HCl (pH 7.5) and lysed by sonication. Cell debris was pelleted at 14,000 rpm over 30 minutes at 4°C, and the supernatant was incubated with Ni-NTA resin (Qiagen) for 3 hours at 4°C. The resin was washed 3 times with 300 µl PBS, 10 mM imidazole-HCl (pH 7.5). The FLAG-4Cys-CaM-hexa-histidine was eluted with 300 µl PBS containing 500 mM imidazole-HCl (pH 7.5).

The protein was labeled with 50 µM CrAsH from a 10 mM DMSO stock in elution buffer at 4°C overnight and excess CrAsH and imidazole were removed via buffer exchange to 50 mM HEPES-NaOH (pH 7.5) and 250 mM NaCl. The solution was concentrated 10-fold on 3 kDa Amicon Ultra centrifugal filters (Merck Millipore). Labeled CrAsH-CaM was further purified by SEC, using a S75 10/300 GL analytical column (GE Healthcare) in a buffer containing 50 mM HEPES-NaOH (pH 7.2), 250 mM NaCl, 0.1 mM CaCl2 and 0.05 mM TCEP.

Fluorescence anisotropy of Ca2+/CrAsH-CaM at a dilution of 3.5 nM was measured in 150 µl of the same buffer completed with 0.1% BSA against a serial dilution of DAPK2 CD+ARD in bio-one low profile flat bottom black plates (Greiner) using an excitation wavelength of 470 ±5 nm and an emission wavelength of 540 ±10 nm in a plate reader (TECAN, Infinite M1000) at 30°C. Each mutant was measured in three different experiments each in triplicate. Each protein mutant was obtained from a single protein preparation batch. Bmax values (plateau height) and K_D's were estimated using a non-linear regression assuming one site-specific binding with the software Prism (Version 5.0, GraphPad). Delta anisotropy values were obtained by subtracting anisotropy values of Ca^{2+}/CaM to anisotropy values of DAPK2 mutant Ca^{2+}/CaM complexes in each experiment. Error bars represent one standard deviation for the representative experiment displayed in figure 5C.

Cellular blebbing assay

For cellular blebbing assays, hDAPK2 FL and hDAPK2 CD+ARD were cloned using XhoI and SalI sites in pECFP-C1 (N-terminal eCFP). Site-directed mutagenesis was used to generate hDAPK2 mutants. HEK293T Cells were grown and seeded as described above. Cells were transfected with 0.4 µg of DNA for blebbing assays and 1 µl Lipofectamine 2000™ (Invitrogen), according to manufacturer instructions. Cellular activity of hDAPK2 through MLC-2 phosphorylation was determined with a plasma membrane blebbing assay (Bialik et al., 2004; Bovellan et al., 2010; Inbal et al., 2002). Live cells were imaged 48 hours post-transfection with a 25-fold magnification objective on an AxioObserver Z1 (Carl Zeiss MicroImaging) inverted fluorescence microscope. Where indicated, cells were treated with 2.5 mM thapsigargin (TG) for 5 minutes at 37°C before imaging. Images were processed with ImageJ (1.47v, Wayne Rasband). Cells were scored manually into either a blebbing or normal phenotype. All experiments were carried out in triplicates.

Table S1 (related to Figure 1): Structural conservation of hDAPK homodimerization.

RMSD values for monomers (Å)

	**hDAPK1**	**hDAPK2 (A/B)**[a]	**hDAPK2 (C/D)**[a]	**hDAPK3**
**hDAPK1**	*0.25*[b]	0.53 ± 0.01 (4)[c]	0.54 ± 0.01 (4)[c]	1.07 (2)[c]
**hDAPK2(A/B)**	1.19	*0.19*[b]	0.18 ± 0.02 (4)[c]	0.65 (2)[c]
**hDAPK2(C/D)**	1.54	0.39	*0.15*[b]	0.67 (2)[c]
**hDAPK3**	1.96	1.68	1.48	ND[d]

RMSD values for dimers (Å)

Legend Table S1: The root-mean-square deviation (RMSD) values of superimposed DAPK heterodimers are about twice as high as those of the superimposed monomeric protein chains in different combinations. For comparison, monomers from identical DAPKs superimpose with < 0.3 Å RMSD (in italics), thus providing a realistic estimate of experimental coordinate errors. Obviously, the elevated values of superimposed dimers from different DAPKs reflect the varying resolution limits of the structures determined and unrelated crystallization conditions that have led to different crystal lattice constrains.

a) Chain labels

b) RMSD's of monomers of homodimers (in italics)

c) Number of measurements

d) ND, no independent refinement of the two monomers

Table S2 (related to Figures 1 and 3): Shared hDAPK dimer interface properties.

	hDAPK1	hDAPK2 (A/B)	hDAPK2 (C/D)	hDAPK3
**Overall interface**				
Total interface area [$Å^2$] [a]	931	1350	1391	1613
Interface residues	29	39	40	49
Polar interactions (< 3.5 Å)	1	18	19	16
**Ca^{2+}/CaM overlapping surface areas**				
CD surface patch I [$Å^2$] [b]			7	48
CD surface patch II [$Å^2$] [b]	29	324	376	376
CD surface patch III [$Å^2$] [b]			12	115
CD surface patch IV [$Å^2$] [b]	318	407	414	424
**CD Surface patches total [$Å^2$]**	**359**	**848**	**968**	**963**
ARD Surface patch V [$Å^2$]	12	117	159	
**All surface patches I-V [$Å^2$]**	**371**	**965**	**1127**	**963**
**Percentage dimer interface**	**40%**	**71%**	**81%**	**60%**

a) Average value of the dimer surfaces contributed by two monomers

b) For details, see Figure 2

Table S3 (related to Figure 4): SAXS analysis of distributed hDAPK2 association states.

	**hDAPK2**	**D220K**	**BL**
**% monomer**	*51 ± 1*	68 ± *1*	89 ± *1*
$\chi^2_{\mathbf{monomer}}$	*1.6*	1.3	1.0
$\chi^2_{\mathbf{dimer}}$	1.6	*2.6*	4.1
$\chi^2_{\mathbf{equilibrium}}$	1.0	1.0	1.0

Legend Table S3: % monomer, the percentage by volume of monomeric DAPK2 estimated by linear least squares fitting in the program OLIGOMER (15); $\chi^2_{equilibrium}$, discrepancy (ie. fit) between the model and experimental data for the equilibrium calculated using OLIGOMER; $\chi^2_{monomer}/\chi^2_{dimer}$, discrepancies for the direct fits of monomeric DAPK2 or dimeric DAPK2 to the experimental data using CRYSOL (PDB ID. 2A2A).

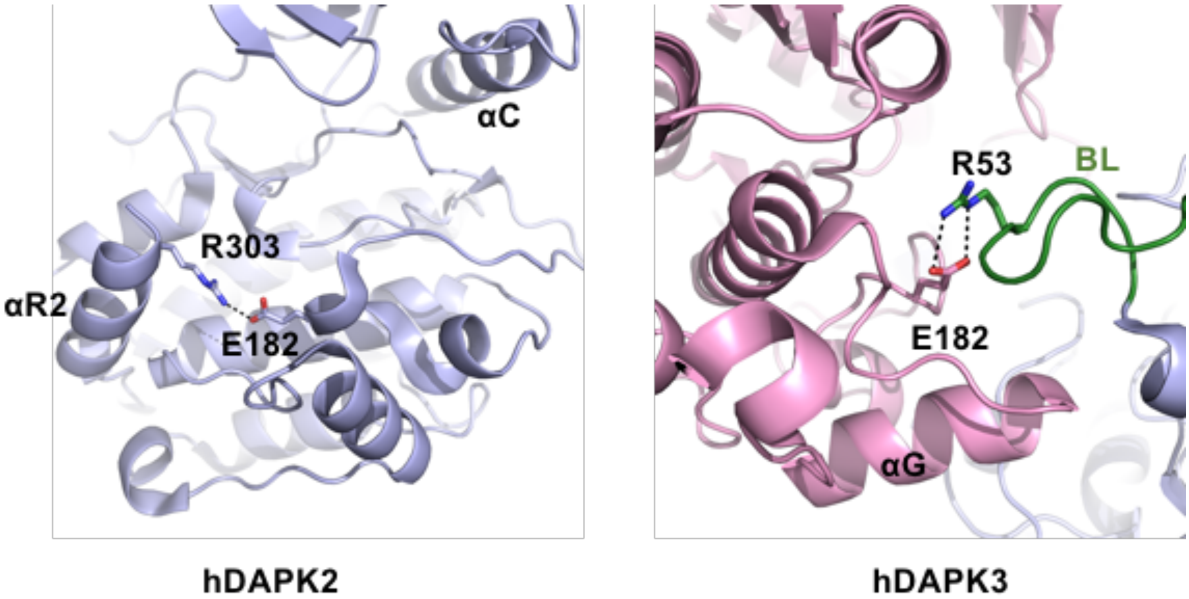

Figure S1 **(related to Figure 3):** Blocking of substrate recognition residue Glu182 by Arg303 from the ARD in hDAPK2 (PDB code 2A2A) and R53 of the opposite monomer BL in DAPK3 (PDB code 1YRP). Hydrogen bonds are represented by black dots. Similar color code as figure 1 has been used.

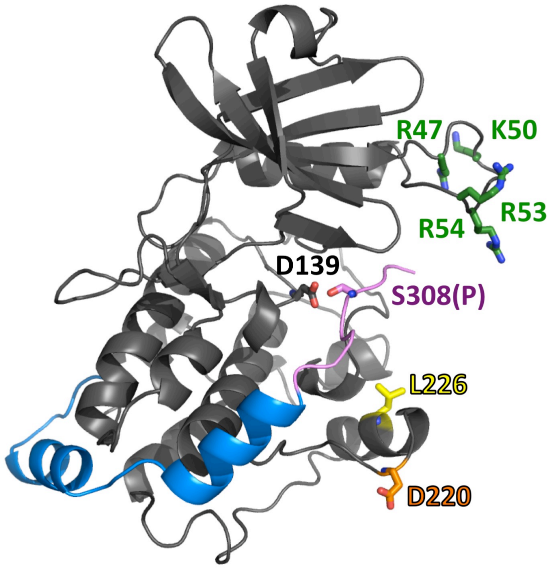

Figure S2 **(related to Figure 2):** Location of DAPK residues mutated in this work with respect to the DAPK overall fold. The coordinates of an extended DAPK1–ARD model (Temmerman et al., 2014) were used to display the relation of these residues to the DAPK active and substrate binding site. The catalytic residue Asp139 is also shown as active site reference. Color codes are as in all other structural figures. The ARD pseudosubstrate segment (residues 303-311) is shown in violet.

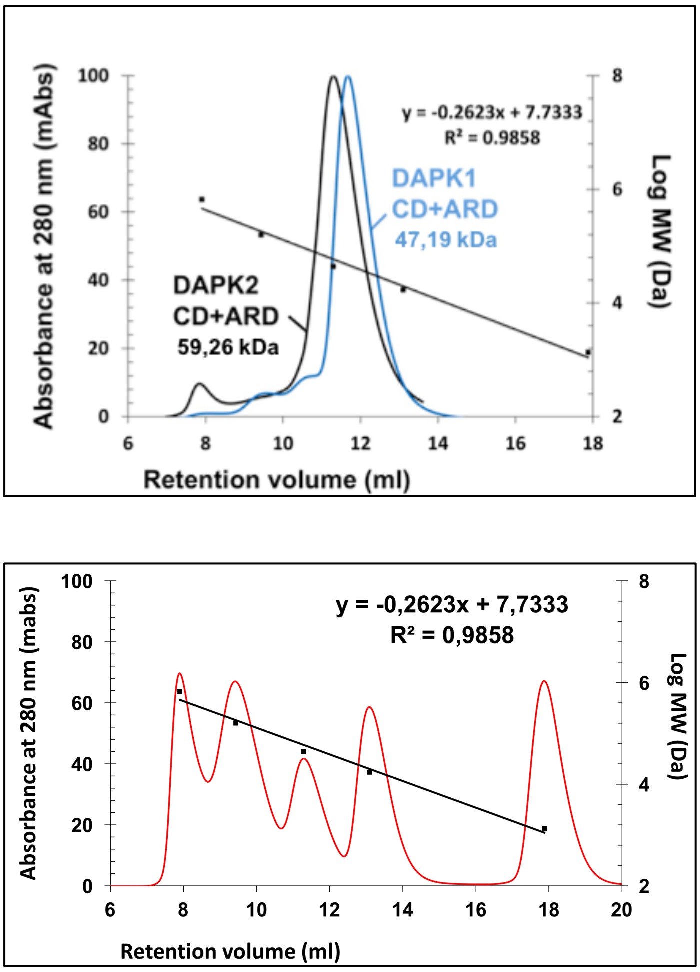

Figure S3 **(related to Figure 4):** Size exclusion chromatography of hDAPK1 CD+ARD and its comparison to hDAPK2 CD+ARD (upper panel). The equation corresponds to the linear fit of the standard proteins from lower panel (from left to right: Thyroglobulin 670000 Da, γ-globulin 158000Da, Ovalbumin 44000 Da, Myoglobin 17000 Da, Vitamin B12 1350 Da). The correlation coefficient (R^2) describes the fit of the linear equation to the standard points. Indicated molecular weights have been calculated from samples retention volume and the equation of the linear fit of standard proteins.

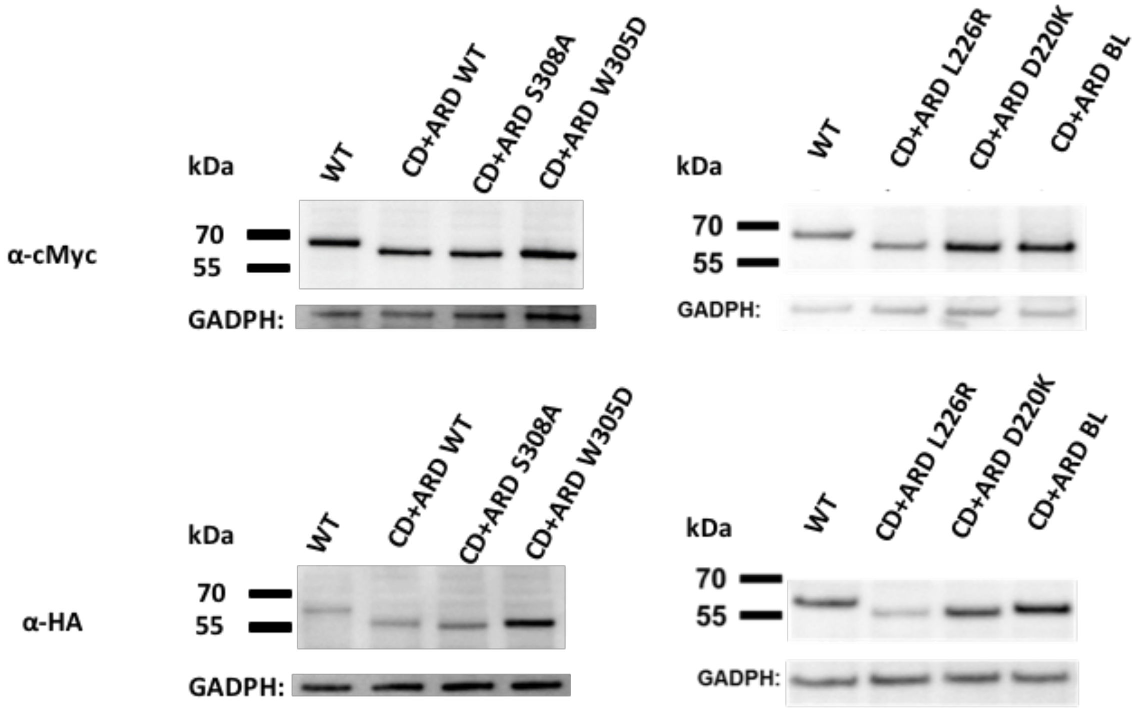

Figure S4 **(related to Figure 4)**: Western blot of BiFC experiment showing expression level of each construct. Western blot analysis of BiFC Venus N-terminal (anti c-Myc tag antibody) and Venus C-terminal (anti HA tag antibody) construct expression level in HEK293T cells. Anti GADPH was used as a loading control.

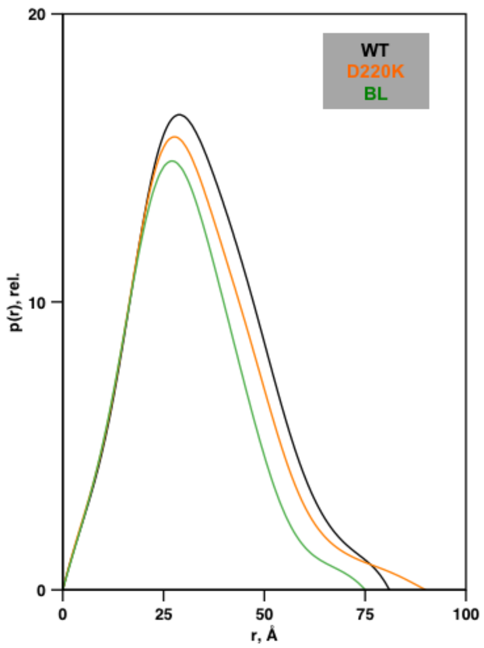

Figure S5 **(related to Figure 4):** Small Angle X-ray Scattering real-space distance distributions, p(r) curves of hDAPK2 CD+ARD variants.

References:

Adams, S.R., Campbell, R.E., Gross, L.A., Martin, B.R., Walkup, G.K., Yao, Y., Llopis, J., and Tsien, R.Y. (2002). New biarsenical ligands and tetracysteine motifs for protein labeling in vitro and in vivo: synthesis and biological applications. J Am Chem Soc *124*, 6063-6076.

Bialik, S., Bresnick, A.R., and Kimchi, A. (2004). DAP-kinase-mediated morphological changes are localization dependent and involve myosin-II phosphorylation. Cell Death Differ *11*, 631-644.

Bovellan, M., Fritzsche, M., Stevens, C., and Charras, G. (2010). Death-associated protein kinase (DAPK) and signal transduction: blebbing in programmed cell death. FEBS J *277*, 58-65.

Cao, H., Chen, B., Squier, T.C., and Mayer, M.U. (2006). CrAsH: a biarsenical multi-use affinity probe with low non-specific fluorescence. Chem Commun (Camb), 2601-2603.

Chen, V.B., Arendall, W.B., 3rd, Headd, J.J., Keedy, D.A., Immormino, R.M., Kapral, G.J., Murray, L.W., Richardson, J.S., and Richardson, D.C. (2010). MolProbity: all-atom structure validation for macromolecular crystallography. Acta Crystallogr D Biol Crystallogr *66*, 12-21.

Emsley, P., Lohkamp, B., Scott, W.G., and Cowtan, K. (2010). Features and development of Coot. Acta Crystallogr D Biol Crystallogr *66*, 486-501.

Evans, P. (2006). Scaling and assessment of data quality. Acta Crystallogr D Biol Crystallogr *62*, 72-82.

Griffin, B.A., Adams, S.R., and Tsien, R.Y. (1998). Specific covalent labeling of recombinant protein molecules inside live cells. Science *281*, 269-272.

Inbal, B., Bialik, S., Sabanay, I., Shani, G., and Kimchi, A. (2002). DAP kinase and DRP-1 mediate membrane blebbing and the formation of autophagic vesicles during programmed cell death. J Cell Biol *157*, 455-468.

Jones, T.A., Zou, J.Y., Cowan, S.W., and Kjeldgaard, M. (1991). Improved methods for building protein models in electron density maps and the location of errors in these models. Acta Crystallogr A *47 (Pt 2)*, 110-119.

Kabsch, W. (2010). Xds. Acta Crystallogr D Biol Crystallogr *66*, 125-132.

Konarev, P.V., Volkov, V.V., Sokolova, A.V., Koch, M.H.J., and Svergun, D.I. (2003). PRIMUS: a Windows PC-based system for small-angle scattering data analysis. Journal of Applied Crystallography *36*, 1277-1282.

Kursula, P. (2004). XDSi: a graphical interface for the data processing program XDS. Journal of Applied Crystallography *37*, 347-348.

Leslie, A.G. (2006). The integration of macromolecular diffraction data. Acta Crystallogr D Biol Crystallogr *62*, 48-57.

McCoy, A.J., Grosse-Kunstleve, R.W., Adams, P.D., Winn, M.D., Storoni, L.C., and Read, R.J. (2007). Phaser crystallographic software. J Appl Crystallogr *40*, 658-674.

Murshudov, G.N., Vagin, A.A., and Dodson, E.J. (1997). Refinement of macromolecular structures by the maximum-likelihood method. Acta Crystallogr D Biol Crystallogr *53*, 240-255.

Perrakis, A., Morris, R., and Lamzin, V.S. (1999). Automated protein model building combined with iterative structure refinement. Nat Struct Biol *6*, 458-463.

Petoukhov, M.V., Franke, D., Shkumatov, A.V., Tria, G., Kikhney, A.G., Gajda, M., Gorba, C., Mertens, H.D., Konarev, P.V., and Svergun, D.I. (2012). New developments in the program package for small-angle scattering data analysis. J Appl Crystallogr *45*, 342-350.

Rutkowska, A., Haering, C.H., and Schultz, C. (2011). A FlAsH-based cross-linker to study protein interactions in living cells. Angew Chem Int Ed Engl *50*, 12655-12658.

Shani, G., Henis-Korenblit, S., Jona, G., Gileadi, O., Eisenstein, M., Ziv, T., Admon, A., and Kimchi, A. (2001). Autophosphorylation restrains the apoptotic activity of DRP-1 kinase by controlling dimerization and calmodulin binding. EMBO J *20*, 1099-1113.

Svergun, D., Barberato, C., and Koch, M.H.J. (1995). CRYSOL - a Program to Evaluate X-ray Solution Scattering of Biological Macromolecules from Atomic Coordinates. Journal of Applied Crystallography *28*, 768-773.

Temmerman, K., de Diego, I., Pogenberg, V., Simon, B., Jonko, W., Li, X., and Wilmanns, M. (2014). A PEF/Y substrate recognition and signature motif plays a critical role in DAPK-related kinase activity. Chem Biol *21*, 264-273.

Tereshko, V., Teplova, M., Brunzelle, J., Watterson, D.M., and Egli, M. (2001). Crystal structures of the catalytic domain of human protein kinase associated with apoptosis and tumor suppression. Nat Struct Biol *8*, 899-907.

Vagin, A., and Teplyakov, A. (2010). Molecular replacement with MOLREP. Acta Crystallogr D Biol Crystallogr *66*, 22-25.
